# “I don't feel fully prepared”: a qualitative study of recently graduated students' mental health experiences of the transition out of university

**DOI:** 10.1002/jad.12431

**Published:** 2024-10-22

**Authors:** Megan J. Magier, Madelyn Law, Tanya Martini, Sarah Pennisi, Kristen M. Lucibello, Karen A. Patte

**Affiliations:** ^1^ Faculty of Applied Health Sciences Brock University St. Catharines Ontario Canada; ^2^ Faculty of Social Sciences Brock University St. Catharines Ontario Canada; ^3^ Student Services and Wellness Niagara College Welland Ontario Canada

**Keywords:** emerging adulthood, psychological well‐being, school‐to‐work, service use, transitions

## Abstract

**Objective:**

This study aimed to better understand the mental health experiences of students as they prepared to transition out of university.

**Participants:**

Participants included 18 recently graduated students from a Canadian university.

**Methods:**

Virtual one‐on‐one semi‐structured qualitative interviews were conducted and analyzed following the protocol for content analysis and using QSR NVivo.

**Results:**

Four main themes were identified, including: distress and feelings of doubt, the importance of connections, the impact of the COVID‐19 pandemic, and experiences with mental health service use. Participants discussed feeling pressured to succeed and a fear of failure, uncertainty and unpreparedness for next steps, the importance of connections to peers and professors, a lack of motivation and feeling ‘unfinished’ due to the COVID‐19 pandemic response, and the need for flexible and accessible mental health services to address immediate and longer‐term needs.

**Conclusion:**

Results have implications for better support of students as they prepare for graduation.

## 1 INTRODUCTION

A developmental gap has emerged in industrialized nations between the end of adolescence and the beginning of adulthood, known as emerging adulthood (18–29 years) (Arnett et al., 2014; MacDonald et al., 2018). Due to social and economic shifts, most individuals now pursue postsecondary education before entering the workforce and/or starting a family. This stage corresponds to the age range when the prevalence of most mental illnesses peaks (Arnett et al., 2014). Emerging adults report higher rates of anxiety and depression than any other developmental period (Chrikov et al., 2020; Larcombe et al., 2016; McGorry & Mei, 2018) and yet, young people are unlikely to use mental health services until they are in a crisis (Alonso et al., 2004; Arnett et al., 2014; MacDonald et al., 2018). Experiencing poor mental health during this stage can hamper the transition into adulthood (Chrikov et al., 2020; Larcombe et al., 2016; McGorry & Mei, 2018), and the recent COVID‐19 pandemic may have increased mental health concerns for this group as they graduated during a period of uncertainty, increased costs of living, and heightened psychological stress (Chrikov et al., 2020). Further, academic study has the potential to have a further negative impact on students' wellbeing (e.g., experiencing pressure to perform well and a competitive environment) (Larcombe et al., 2016; McGorry & Mei, 2018). Therefore, there is a crucial need to address the mental health of emerging adults. While research has examined mental health as students graduate secondary school and transition into postsecondary education (Carver et al., 2015; Hallett et al., 2020), there is lack of research focusing on the next major transition in many young people's lives; that is, as they graduate from postsecondary degrees.

Success during the transition out of university is often characterized by employment with well‐being found to decrease with increasing durations of unemployment (Huegaerts et al., 2020). Emerging adults with higher self‐esteem find employment more rapidly, and having a job during higher education has positive effects on career trajectories (Huegaerts et al., 2020). However, higher education is not always directed towards developing the practical skills necessary for the workplace (Grosemans et al., 2020). A mismatch exists between the education required for work and the prior experience required by the employer, and this discrepancy can prolong the period of unintended unemployment (Huegaerts et al., 2020).

Interventions to support emerging adults during their transition out of university are lacking. The available resources in one's environment are important predictors of success; however, equity‐deserving populations (e.g., gender diverse, lower socioeconomic status, and racialized populations) tend to face decreased access to services (Huegaerts et al., 2020; MacLeod & Brownlie, 2014). Practical supports for this age group can include transportation, financial support, and/or health insurance (Wood et al., 2017). Previous research has also shown that emerging adults exhibit the highest drop‐out rates and the lowest uptake rates from mental health services (Carver et al., 2015). The transition after graduating from postsecondary education may be a particularly sensitive period for connecting emerging adults to services as they often lose access to the mental health care they were receiving within the campus setting. Emerging adults may graduate out of parental, student, and government health benefits, and as a result, represent one of the largest age groups without health insurance (Collins et al., 2005). There is a paucity of research that specifically explores the mental health and mental health service use experiences of graduating students as they move through this transition. To date, most research has focused on the transition from secondary education into postsecondary education. Research that informs the development of mental health supports that are deemed relevant from graduating students themselves is also lacking to ensure uptake of services.

Given the limited literature, particularly in the current context and since the onset of the COVID‐19 pandemic, this qualitative study aimed to explore the shared and unique mental health experiences of students as they prepared to transition out of university. In doing so, this research has the potential to advance our understanding of this critical period by focusing on students' perceptions of the key challenges and facilitators to positive transitions. As such, it may help inform interventions to better support emerging adult mental health. The following research questions were explored:

1. What were students' mental health experiences during the final year of their undergraduate degrees as they prepared to transition out of university?
2. What challenges did they experience during the final year of their undergraduate degrees as they prepared to graduate?
3. What did students find helpful during the final year of their undergraduate degrees as they prepared to graduate?

## 2 METHODS

This study used qualitative data from the *Student Health in Final‐year Transitions (SHIFT) Study*. Ethics approval was obtained from the institutional Research Ethics Board (#20‐271) at Brock University.

### 2.1 Theoretical framework

Guided by the Life Course Theory (LCT), the current study focused on the transition out of postsecondary education and the social and cultural factors that influence how this transition is navigated. The LCT theory focuses on trajectories—long‐term patterns in individuals' lives—and how earlier life impacts later health and success, placing development in cultural and historical contexts (Halfon et al., 2018; Hutchison, 2011). Transitions are understood as discrete and bounded social role or status changes that are embedded in trajectories (Hutchison, 2011). How individuals navigate these transitions is imperative to their success.

Emerging adulthood has been cited as a critical developmental stage in the life course (Wood et al., 2017). Developmental trajectories that appear during emerging adulthood depend upon multiple biological, social, cultural, and physical factors (Wood et al., 2017). Navigating challenges that arise during emerging adulthood will inherently influence the outcomes in the adult stage of life (Wood et al., 2017). According to Arnett, individuals in this stage of life experience physical and sexual maturity, and have optimism towards educational and occupational trajectories, but are still experiencing heightened instability, identity exploration, self‐focus and feeling in‐between (Arnett et al., 2014). Together, the LCT and emerging adulthood were used as frameworks to advise the development of the interview guide. Questions focused on challenges, supports, and impacts on mental health to explore how to support positive transitions out of university.

### 2.2 Participants

Students who participated in the April 2021 (Year 1) and 2022 (Year 2) survey from the S*tudent Health in Final‐year Transitions (SHIFT) Study (*Magier et al., 2023 *)* and expressed interest in participating in an interview at the end of the survey were eligible to participate. Eligible participants all attended the same public university in Southern Ontario, Canada. This university is considered mid‐sized relative to other Canadian universities (2021/2022 enrollment = 17,000 undergraduate students). Survey participants who indicated interest were sent a follow‐up email to invite them to participate in an interview. A link to an online Qualtrics survey was sent asking initial screening questions to ensure variation by gender, age, career/education status, and faculty was present in the sample. Sociodemographic information for each participant was asked in an online Qualtrics survey before the start of the interview (see Table 1). Most participants identified as cis‐women and white, and were in a Health Sciences faculty, currently working (paid employment/entrepreneurship), living with family, and born in Canada. The average age was 23.8 (standard = 1.06) years.

**Table 1:** Demographic information for graduated students that participated in an interview for the SHIFT Study (*N* = 18).

Demographics		*N* (%)
**Faculty**	Education	2 (11.1%)
	Social Sciences	4 (22.2%)
	Health Sciences	7 (38.9%)
	Mathematics and Science	1 (5.6%)
	Business	4 (22.2%)
**Age**	Mean [SD]	23.8 [1.06]
**Current career/education status (check all that apply)**	Enrolled in a graduate/professional university degree	7 (38.9%)
	Working (paid employment/entrepreneurship)	12 (66.7%)
	Deciding what to do next	1 (5.6%)
**Gender**	Cis‐Women	11 (61.1%)
	Cis‐Men	5 (27.8%)
	Transgender and/or Genderqueer	2 (11.1%)
**Race/Ethnicity**	Arab	1 (6.3%)
	Black	1 (6.3%)
	Korean	1 (6.3%)
	South Asian	3 (18.8%)
	White	10 (62.5%)
**Relationship Status**	Single	8 (44.4%)
	In a relationship	9 (50.0%)
	Married, in domestic partnership, or engaged	1 (5.6%)
**Living Arrangement**	With family (e.g., parents, spouse/partner, children)	15 (83.3%)
	Alone	3 (16.7%)
**Born outside of Canada**	Yes	5 (27.8%)
	No	13 (72.2%)
**Parent(s)/guardian(s) born outside of Canada**	Yes	13 (72.2%)
	No	5 (27.8%)
**Highest formal education obtained by a parent/guardian**	High school or less	1 (5.6%)
	College program	5 (27.8%)
	University degree (e.g. Bachelor's)	9 (50.0%)
	Graduate or professional degree	3 (16.7%)
**Current Financial Situation**	Often stressful	1 (5.6%)
	Sometimes stressful	10 (55.6%)
	Rarely/Never stressful	7 (38.9%)
**Financial Situation Growing up**	Often stressful	4 (22.2%)
	Sometimes stressful	6 (33.3%)
	Rarely/Never stressful	8 (44.4%)

Sample size was guided by the data analysis and interpretation, as well as time limitations and practical implications of the data collection period. Following initial analyses and interpretation of the data, sampling continued until no new information was acquired (Fusch & Ness, 2015). By this time, 18 participants were interviewed and after review of similar qualitative studies (Koo et al., 2021; Perry, 2012), this quantity was deemed a reasonable ending point.

### 2.3 Data collection

Data collection took place from March to May of 2023. Virtual one‐on‐one semi‐structured interviews were conducted by the first author (MM) with participants using Microsoft Teams (a videotelephone software). Interviews were used to gain insight into individuals' lived experiences and understand how individuals' experiences relate to other study participants through data analysis (Ravitch & Carl, 2022). A semi‐structed interview guide was used for flexibility, consistency, and comparability, and to produce rich, detailed data (Braun & Clarke, 2013). Interviews lasted between 30 and 60 min. Participants were compensated with a $20 e‐gift card.

### 2.4 Measures

The interview guide (Appendix 1) was developed by the research team, including a final‐year undergraduate student representative, to explore student mental health and access to services and supports in the year before graduation. The interview guide was then reviewed by four fourth‐year undergraduate students to ensure the relevance, clarity, and comprehensiveness of the questions. The interview was pilot tested with other students to ensure clarity and comprehensiveness of the questions. Changes were made to the interview guide as needed, and then data collection began. Open‐ended interview questions were asked, such as “How did your experiences in preparing to graduate relate to your mental health?” followed by probing questions to provide further details.

### 2.5 Coding procedures

Interviews were audio recorded and transcribed verbatim using Microsoft Teams and checked for accuracy by MM. Analysis followed the protocol for content analysis using QSR NVivo (Bengtsson, 2016). Content analysis involves examining language and classifying large amounts of text into categories that sufficiently represent similar meanings to produce knowledge and understanding of the topic under study (Downe‐Wamboldt, 1992; Weber, 1990).

This process began with a familiarization phase, during which the first author read each transcript in its entirety, reread the transcript to gain an in‐depth understanding of the content, and then created meaning units on content in which participants provided meaningful indications of their experiences, inductively. Next, meaning units were read and categorized based on patterns, and then codes were organized into realistic themes. Themes were refined with the research team. MM acted as the coder for this analysis. An example of the analysis process is included in Table 2. Consistent with the process of inductive content analysis, results were shared with others who have familiarity with the research topic, which included co‐authors of this paper (Elo et al., 2014). Co‐authors were mentors to the first author and this research project and have expertise in mental health, emerging adulthood and qualitative methods. KAP was the primary supervisor and Principal Investigator of the SHIFT study, with her own experience of working in and accessing mental health services. SP was the Director of the university student mental health services. In addition, four recently graduated students—who varied sociodemographic factors such as gender and ethnicity—who had assisted in the development of the interview guide were also involved as critical friends, to ensure codes and themes stayed true to graduating emerging adult experiences, and to challenge the authors' assumptions. It is important to note that the critical friends involved all had their own final‐year experience in their undergraduate degrees and may have also had their own mental health and mental health service use experiences that shaped their participation in this research.

**Table 2:** An example of the analysis process using transcribed interviews with graduated students to explore student mental health and access to services during their final year of their undergraduate degree.

Meaning unit	Condensed meaning unit/Simplified expression	Code	Subcategory	Theme
“…and in undergrad, I get sad that it was so **isolated**. I'm just checking in and make sure everyone's doing OK, but I think the **loss of that face‐to‐face interaction** with your professors and your peers **was really hard**.”	Felt isolated during final year Difficulty connecting with others	Isolated Social disconnection Difficulty connecting COVID‐19	Feeling disconnected from peers due to the COVID‐19 pandemic	Importance of connections

## 3 DATA RIGOR

To enhance the ‘trustworthiness’ of this study, working with critical friends was used to stimulate discussion, provide feedback, and encourage reflexive acknowledgement of results. Involving critical friends adds to the reliability of the study, as issues with credibility and trustworthiness have been recently cited within the literature for other techniques, such as member checking (Smith & McGannon, 2018). Participants are quoted in the results section to provide examples of how categories and themes were derived. As mentioned, nonparticipant members of the population of interest (i.e., recent graduates of undergraduate degree programs) were invited to provide feedback and discussion of results, to further challenge author assumptions and ensure analysis appropriately reflected the experiences of graduating students. Rigor was sought in terms of a collaborative research process to achieve richer interpretation of meaning, thus, discussions with critical friends included assessing codes for relevancy, areas of discrepancy and agreeance, as well as ensuring there was cohesiveness within themes (internal homogeneity) and distinctions between themes (external heterogeneity) (Braun & Clarke, 2013).

MM kept analytical notes during the analysis (e.g., regarding reasoning and reflections) to maintain reflexivity and a non‐judgemental interview approach was used to limit the influence of predispositions on research findings (Goodell et al., 2016). MM also reflected on preconceived assumptions throughout the analysis, consistent with the practice of reflexivity (Berger, 2015).

### 3.1 Positionality

This study adopts a social constructivist perspective which acknowledges that every individual brings their own experiences and that multiple views of reality exist (Tamminen & Poucher, 2020). Knowledge is created between the researcher and the participant, where the researcher attempts to interpret those experiences and meanings that others have about the world (Tamminen & Poucher, 2020).

As a female doctoral candidate, my interest in mental health and emerging adulthood stemmed from my own experiences. Moving from my undergraduate degree into further postsecondary education, I fit within the features of emerging adulthood (Arnett, 2015). Currently pursuing further education and delaying entrance into the workforce/marriage, I also have been experiencing identity exploration, self‐focus, feeling‐between and possibilities/optimism (Arnett, 2015). Moreover, my experiences during emerging adulthood have affected my mental health. Having sought my own mental health care, experiencing delays in wait times, heightened anxiety, and the COVID‐19 pandemic, my interest in what others were experiencing increased. I am privileged to have a therapist who acknowledges my values and beliefs and to have family, friends, and mentors who recognize the value of mental health care. However, I recognize that others have different experiences. I would like to be able to understand the perspectives of others as they transition from university to create better outcomes for everyone.

As I move through each stage of this research, I acknowledged my own experiences, beliefs, and values by actively engaging in reflexivity. Reflexivity is a process through which qualitative researchers' acknowledge that their own biases, values, and ways of understanding influence the research process (Creswell & Poth, 2023). While creating the research questions, I consistently referred to my theoretical frameworks of the LCT and emerging adulthood to ensure the research questions were adding to the literature in a significant way. Since my experiences have the potential to shape my coding, categorizing, conclusions and interpretations of the data, I kept analytical memos to reflect upon my interactions with the data. Moreover, using critical friends helped limit my own biases that became present when contradictions and/exceptions within the data arose. Details regarding my own previous experiences with mental health and mental health service use were not shared with the other authors or critical friends, to avoid potentially influencing their interpretation of the data.

## 4 RESULTS

The findings describe experiences that students had during the final year of their undergraduate degree programs while they prepared to transition into their next steps. The four main themes that students expressed included: distress and feelings of doubt, the importance of connections, the impact of the COVID‐19 pandemic, and their experiences with mental health service use and accessibility. Culturally relevant and gender‐appropriate pseudonyms are used throughout to protect against identification of participants. The most common experiences have been reported below.

### 4.1 Distress and feelings of doubt

The distress and feelings of doubt theme included three main challenges that participants experienced during their final year: feeling pressure to succeed and fear of failure, feeling unprepared and wanting guidance on next steps, and stress regarding the uncertainty of navigating the next steps.

#### 4.1.1 Pressure to succeed and fear or failure

One recurring challenge discussed by participants was the pressure they felt to succeed academically during their final‐year of their undergraduate program and the toll that took on them (n = 6). For instance, Mia said,

*“I could tell that I wasn't taking care of myself, wasn't taken care of physically, mentally, but the pressure of needing to succeed academically was just a constant.”*

Relatedly, interviewees discussed the fear of failing their courses and the impact that would have on their future employment and mental health. Dave said,

*“I was lucky enough to get a full‐time job offer out of my last co‐op term, so my concern was always if I fail courses and I don't end up graduating, is there going to be an issue with me being employed because I don't think they'd want to employ me if I'm not graduated. So that was something that was a big stressor to me and that would get worse. I did struggle a bit with my mental health, especially as the stress started to build and as I got more anxious with the potential of failing.”*

Participants expressed feeling pressure to maintain academic excellence in their final year of their programs to ensure the security of their next steps.

#### 4.1.2 Feeling unprepared and wanting guidance to navigate next steps

Students expressed not knowing what to expect for the transition (*n* = 9). Colin explained,

*“I think I just really was scared as I think a lot of people are in that situation, just the next step in life and adulthood and a career and figuring that all out… You know a place to live and figuring that out with like a new job and what that's going to look like.”*

The participants described feeling unprepared and wanting guidance, and how these experiences impacted their mental health. Sophie shared,

*“My mental health during that year was like the worst that it had ever been. I feel like I had a bit of imposter syndrome because I didn't know. I feel like I didn't know how I got here. I don't know how to do anything anymore. That [guidance] would have been very helpful because I feel like I don't know what I can access and not having as much like access to like my student e‐mail and my student account and stuff like that. I feel like I don't really know where to go and it's kind of like I'm in limbo. I'm kind of lost, you know. … I don't feel like I was fully prepared.”*

#### 4.1.3 Stress regarding the uncertainty of navigating next steps

Many participants spoke about feeling stressed regarding the uncertainty of the transition out of university and navigating what comes next (*n* = 8). Many participants described stress related to trying to finish school while applying for jobs. Sarah explained,

*“…on top of doing assignments and projects you were also trying to apply to and pick a million different jobs being posted to try to figure things out. So, I would say that there was an added stress level in that aspect, like the what's the next step.”*

Most participants discussed feeling unsure about next steps, particularly if they should be applying for more education or trying to enter the workforce. Erin said,

*“It was really stressful because I wasn't even sure if I was going to finish there. There was talk about doing like many different options. Am I going to just stop there and see if I'm going to get a job and just apply and see what happens? Am I going to try to go get a second degree? Should I go to college? Should I get an extra certificate or things like that? So that really stressed me out also.”*

The uncertainty of what to do after their undergraduate program, and the perception that a multitude of pathways exist, became an added stress when trying to finish their courses.

A small subgroup notably discussed how having a job set‐up after graduation contributed to feeling less stress and uncertainty (*n* = 4). Alex said,

*“Like I mentioned I had the job offer in hand from my now previous company. Uh, so being able to like not have to deal with the stress of OK, I've graduated now I need to update my resume. Now I need to go find a job and you start applying and interviewing at places. Like as soon as I finish that last exam and I knew that I passed. Was like, OK, cool, in 2 weeks I start.”*

### 4.2 The impact of the COVID‐19 pandemic

Participants graduated during the COVID‐19 pandemic, and as such, experiences specific to the pandemic context were highlighted in most interviews. Due to the pandemic response, universities were closed to in‐person learning during this time and only offering online classes. Many students discussed the COVID‐19 pandemic as contributing to a lack of motivation during the final year of their program and feeling unfinished given an anticlimactic ending to their undergraduate degree.

#### 4.2.1 Lacking motivation

Some participants described the difficulty in trying to maintain motivation to finish final‐year classes while completing virtual courses, due to the COVID‐19 pandemic response (*n* = 6). Dante explained,

*“I think also finding the motivation to do things, it's hard to be motivated when you're not seeing the professor. You don't have like ‘oh I need to go in person to go to this class’. It's like I could easily just not go to class because I'm just an anonymous face online. Something that was especially for my fourth year was big challenge is kind of general motivation of going to class and getting work done.”*

The majority of participants also explained feeling burnt out while working from home during the COVID‐19 pandemic response, contributing to the lack of motivation. Josie said, “*I felt like I was experiencing the effects of burnout because I was just working myself for so many more hours than I did when I was actually going in person*” and felt “*it was a bit difficult to try to get my groove and trying to you know make a to do list and try to stick to that and keep myself motivated*.”

#### 4.2.2 Feeling unfinished

A smaller portion of participants mentioned missing out on having a convocation ceremony due to the COVID‐19 pandemic response (*n* = 5). Interviewees discussed not being able to celebrate their accomplishments and feeling unfinished as having an impact on their final‐year experience. Dante explained, *“Like it kind of just fizzles out, so didn't feel like an ending, just felt like I'm just going to do something else…”* Madi explained her experience by saying,

*“I feel like I would have felt different if things weren't like the scenario with the pandemic and everything as it was… I feel like I would have felt more accomplished and happy if like there was actually like a celebration of the end of graduation or just some sort of like event that.”*

Participants further mentioned feeling like they missed out on other opportunities, Elijah explained, *“my intention about my 4* ^*th*^ *year and 2020 as a whole was that I'm going to go out of my comfort zone a little bit more. I'm gonna make more friends. I'm gonna go out to more events. And I was like. No. OK, so that's not happening.”*

### 4.3 The importance of connections

Recurringly, interviewees discussed the importance of connections during their final‐year of their programs, particularly with their peers and professors.

#### 4.3.1 Feeling disconnected from peers due to the COVID‐19 pandemic

Almost all participants discussed the importance of feeling connected with their fellow students and friends during their final year (*n* = 15). Some students discussed feeling socially disconnected and isolated, due to the online classes during the pandemic response. Sarah explained,

*“I think whatever little mental support I received was from talking with friends. You know, you ran to your friends and like, they would hear you out, give you like maybe a touch of advice. They'll tell you what they're going through. All that was for sure missing [due to COVID], so I did feel a bit isolated there.”*

Other interviewees described the difficulty in building connections while having class online during their final year. Mia said,

*“So, I was at the point in my third year where I just got the courage to really get involved with things on campus and trying to foster those lifelong friendships. You're trying and then it all just became so isolated all the sudden. So, like my 4th year, you tried to connect with people, but it was hard because everyone just kind of doing their own thing.”*

Participants discussed their desire to build on or be able to create new connections with their colleagues during their final year. Michelle described starting to make stronger connections with peers while in the third year of her program,

*“but then we didn't know how long the pandemic was going to be. So, everyone kind of just, like gave up and was like, ‘I'll talk to you when we can’, not realizing that we're never going to kind of connect again. I also felt like I lost a lot of my (program name) friends during that because we thought it was going to be a limited time, but it eventually grew longer and longer. And then by the time we're reaching out to reconnect, it was like we don't really know when or who we should be seeing.”*

#### 4.3.2 Connections with professors can have an important impact

Additionally, interviewees discussed the positive and negative impacts that interactions with professors can have on their final‐year experiences (*n* = 11). Hasan described an example of a negative interaction with a professor when asking for support,

*“going to this conversation with one of my professors and him telling me that roughly one‐third of the students who take this course have to retake it, in a conversation where I was telling him about how my mental health was deteriorating because I felt like I was going to fail the course, was probably not the best thing for him to talk about.”*

Contrarily, when asked what was helpful for students as they prepared to graduate, many described having positive interactions with professors. Colin stated,

*“I think that my professors honestly, because obviously they're teaching like the fourth year classes. They know that we're graduating this year and like we're about to start our jobs and stuff. A lot of them, like, gave a lot of good advice and prepared you kind of for how that would look like or what you should do, and I remember some of them even like offered to write my recommendations or be references when I apply to jobs and stuff, so that like made me feel a lot better that I kind of had that support. Yeah, that helped me a lot.”*

### 4.4 Mental health service use and accessibility

Among interviewees that discussed having accessed formal mental health support in their final‐year, the two primary experiences described were how counseling can have a positive impact and the importance of flexibility in services.

#### 4.4.1 Positive impact of timely access and need for longer‐term support

Of those students that accessed support, some described the value of being able to quickly access mental health services (*n* = 4). Dante explained, “*It is helpful more for like here this immediate thing that I need to chat about for now*,” but continued that this immediate support, “*wouldn't really resolve a problem”* because *“you need kind of like longer form therapy*.” Similarly, Alyssa said,

*“I found the counseling was really helpful because it not only validated the way I was feeling, but also kind of showed you like, other people also feel this way too. And like, there's ways that we can help you combat the way you're feeling. So, it was really positive. I did stick with the (University services) for a few months and then we went to private because it was offered in person from a local person. So, I felt that was just important for me to be able to have that face‐to‐face experience.”*

Interviewees described this quick initial contact with a university provided mental health counselor as useful to discuss their current needs and next steps, but re‐iterated that a longer form of continued therapy was necessary and required going outside of campus‐based services.

#### 4.4.2 Attending appointments as an added stress and need for flexible services

Participants described feeling overwhelmed with scheduling and/or attending appointments (n = 8). They felt that accessing mental health services was a stressor, particularly when experiencing anxiety and/or depression, and added to their already busy schedules. For instance, Gabriel said,

*“They [mental health appointments] kind of overwhelmed me even more I find, and kind of made the issues even worse. So, I didn't personally feel like they were helping that much that you know, when you have to go out of your way to go somewhere, and put it in your agenda, and put in your schedule; for someone who has an anxiety disorder, it kind of just adds (to) the anxiety of everything.”*

Similarly, another interviewee, Madi, explained, “*I felt like I was so busy. If I wasn't doing schoolwork or like trying to finish an assignment, I was either like applying to jobs that I really felt like I didn't have a break to [access therapy].”*

Students shared how flexibility in mental health service delivery (e.g., scheduling of appointments, location) was helpful to reduce the burden of accessing services. Erin said,

*“when I was really depressed, like when I was at my lowest, I wouldn't even go to some of the appointments; I just didn't want to get up there. So, I think having something where there is drop‐in hours, where people don't need to make that commitment, they can just go if they feel like it.”*

## 5 DISCUSSION

This study aimed to better understand the mental health experiences of emerging adults during the final year of their undergraduate degree programs as they prepared to graduate. Attention was paid to perceptions regarding both challenges and facilitators of positive transitions. Four main themes were identified, including: distress and feelings of doubt, the importance of connections, the impact of the COVID‐19 pandemic, and experiences with mental health service use.

Participants in this study discussed feeling pressured to succeed and a fear of failure, feeling unprepared and wanting guidance, and stress regarding the uncertainty of navigating next steps. Students expressed concern with how lower academic marks could impact their future careers prospects and how that pressure ultimately impacted their mental health. Participants mainly discussed feeling pressure from the job market to fall within the top percentile of candidates to secure a career. The academic pressure students face, in combination with work and other life challenges, while exploring and preparing for life after postsecondary education may put students at an increased risk for mental health concerns (Eisenberg et al., 2007; Rudenstine et al., 2021; Yang & Yang, 2022). Participants discussed feeling overwhelmed with the multitude of pathways that exist after graduation and felt unprepared and uncertain about navigating the transition. Most graduates will explore multiple work possibilities, attempting to find the right fit (Marshall & Butler, 2014). Previous research has suggested that emerging adults are accepting of the instability that comes with exploring work possibilities, since they are hoping to find satisfying work (Marshall & Butler, 2014; Murphy et al., 2010). Arnett proposes that exploring possibilities and optimism for the future align with identity development, as emerging adults see their career as an integral part of their identity (Arnett, 2011, 2014). However, participants in this study expressed more concern (i.e., uncertainty, unpreparedness and stress) than optimism for the transition out of postsecondary education. The school‐to‐work‐transition has also become increasingly complex and challenging due to shifts in the labor market (Brzinsky‐Fay & Solga, 2016; Cebulla & Whetton, 2018). Traditionally, this transition was posed as a predictable, structured pathway that individuals could enter and remain with that employer for their entire career (Akkermans et al., 2021). More recently, it is taking graduates longer to find their first job, creating uncertainty as students move into the workforce (Koivisto et al., 2010). Additionally, graduates express feeling unprepared and wish they had more guidance on the school‐to‐work transition (Halstead, 2014). Importantly, individuals who feel uncertain about next steps may become more vulnerable to anxiety and depression (Arnett, 2014). Investigating ways to help students effectively cope with the stress of navigating the workforce may be worthwhile (Huegaerts et al., 2020; Pfeiffer D.). Helping graduates create strategic plans to guide them through the school‐to‐work‐transition may be beneficial in easing the transition (Akkermans et al., 2021; Koivisto et al., 2010). Specifics on the type of guidance graduating students may want to help them navigate the school‐to‐work transition are lacking within the literature. However, internships and other experiences that provide hands‐on learning may be valuable. These programs can help diminish uncertainty by creating connections, confirming or altering career paths, or potentially helping to secure a career (Halstead, 2021). These programs may also help increase self‐esteem and confidence for transitioning emerging adults to find employment more rapidly (Huegaerts et al., 2020).

Participants highlighted the importance of building connections with their peers while preparing to graduate as they could relate to them and normalize each other's experiences. This discussion particularly emerged in relation to the COVID‐19 pandemic measures, including learning online, in that students described feeling disconnected from peers in their final year. Research has shown the COVID‐19 pandemic may have negatively impacted relationships for emerging adults, creating less perceived friend support, and increased loneliness and depressive and anxiety symptoms (Philpot et al., 2021; Rogers et al., 2021). Given that emerging adults place an emphasis on their social support network (Arnett, 2014; Murphy et al., 2010), they may have felt a “developmental mismatch” when many moved back home to live with family, where they were unable to explore new relationships or continue to develop independence (Dotson et al., 2022). Typically, emerging adults' friendships are closely linked to life‐events that occur during this stage, as friendships formed are based on similarities (i.e., hobbies, educational attainment) (McNamara et al., 2015). Postsecondary education and the workplace afford settings to easily make new friends in similar positions (Allan, 2008). Friendships foster autonomy in emerging adults by providing acceptance and encouragement and are an often‐overlooked source of support (Deci et al., 2006; McNamara et al., 2015).

As emerging adults place an emphasis on their peer relationships, the lack of connections students felt during the COVID‐19 pandemic may highlight potential concerns (e.g., increased loneliness, diminished mental health experiences, as highlighted in this study) as they move through their transition. Supporting students to build relationships in pre‐graduation contexts may help them create those primary support networks they feel are lacking (Murphy et al., 2010).

Participants also discussed how connections with their professors can have an important positive or negative impact. Some participants discussed negative interactions they experienced with their professors that led to increased stress for the respective course. Contrarily, some participants discussed having helpful professors (i.e., professors that provided references for jobs, offered job advice and support) that eased their stress regarding next steps. Previous research has shown that faculty‐student interactions can play an important role in student success (Eimers, 2001; Pascarella & Terenzini, 1979; Umbach & Wawrzynski, 2005). Komarraju et al (Komarraju et al., 2010). found that students' academic self‐concept was related to their sense of their professor's approachability, accessibility, and respect for students. Additionally, research has found that the quality and comfort of professor‐student interactions affected the effort students put into their courses (Kuh & Hu, 2001; Micari & Pazoz, 2012). It is possible that professors may underestimate the impact they can have on their students (Micari & Pazoz, 2012).

As participants in this study completed their final year of their undergraduate programs during the ongoing COVID‐19 pandemic, they discussed challenges specific to the response measures. Many participants discussed the lack of motivation they felt in their final year related to shifts to remote learning. Some participants described feeling anonymous online, creating a lack of interaction with the professor and peers, and decreased motivation. Other participants described working for longer hours than when in person, contributing to burnout. Social belonging to their institutions is particularly important for educational success in emerging adults (Marler et al., 2021). Previous research has shown that when students feel disconnected from their institutions, they report less motivation to engage academically (Yeager et al., 2013). Academic motivation and success can provide students with a sense of purpose in one's life, which also may help diminish worry, and enhance resilience to help set up graduates for a positive transition (Marler et al., 2021; Schaefer et al., 2013). Importantly, research on academic motivation and emerging adulthood has mainly focused on the transition from high school to first‐year university, rather than students transitioning out of postsecondary education (Grabau, 2024; Marshall & Symonds, 2021; Kindelberger, Safont‐Mottay, Lannegrand‐Willems & Galharret, 2020). Further research exploring academic motivation and the transition out of postsecondary education is needed. As lack of motivation is a concern as remote courses continue and are increasingly common. Interventions to increase students' sense of belonging within institutions may help increase academic motivation, which in turn, may increase their sense of purpose and general well‐being (Lambert et al., 2013; Marler et al., 2021; Schaefer et al., 2013).

Participants discussed how not having a definitive ending point in their postsecondary education made them feel unfinished. Due to the COVID‐19 pandemic, convocation ceremonies were completed online, and students expressed longing for the celebration that typically comes with convocation. Participants also described experiences of feeling in‐between school and their next steps in life (i.e., on the way to adulthood, but not there yet), which aligns with Arnett's description of emerging adulthood (Arnett, 2014). For most individuals, an event, such as completing education and/or being in full‐time employment, is an important marker of the transition into adulthood (Hutchison, 2011; Johnson et al., 2007; Molgat, 2007). Lacking an important marker of the transition may impact graduates' well‐being and contribute to feelings of depression and anxiety (Arnett, 2015; Johnson et al., 2007). As this finding was specific to the COVID‐19 pandemic response and canceled convocation, feeling unfinished may not be as applicable to emerging adults outside of this context. Research exploring post‐pandemic graduation experiences and the meaning of convocation ceremonies may prove valuable to informing strategies to promote positive transitions.

Finding ways to increase engagement and uptake with service use for emerging adults is necessary to increase retention, as they are more likely to disengage from treatment compared to any other age group (Alonso et al., 2004; Chrikov et al., 2020; Larcombe et al., 2016). Our results highlighted areas of improvement in mental health service access for final‐year graduating students. First, the participants that had accessed mental health services, discussed how having quick initial contact was helpful to provide immediate support, but that this support needed to be followed by longer‐term therapy. Prior research indicates that long wait times for access to services can increase emotional distress, reduce functioning, and exacerbate existing symptoms (Brown et al., 2023; Osuna, 1985). One suggestion that has been proposed is the Stepped Care Approach (Mental Health Commission of Canada, 2021), in which the most effective and least resource‐intensive treatment is offered first. Using this approach, students are seen on a walk‐in basis and provided with an initial contact, and then a treatment plan based on need is developed (Centre for Innovation in Campus Mental Health, 2019). Although further research should investigate counseling supports for graduating students, the Stepped Care approach offers a promising approach to meeting the needs that emerging adults stated.

Some participants discussed the added burden they felt in having to schedule and attend mental health services, while already feeling overwhelmed with other responsibilities. Most participants who had tried to access mental health supports explained the need to provide flexibility regarding scheduling (i.e., offering evening appointments and drop‐in sessions), location (i.e., not having to commute) and mode (i.e., online vs. in‐person) to meet the varying needs of students. Previous research has also reported the availability, location, and timing of appointments as barriers to students accessing support (Stebleton et al., 2014). Increasing availability and types of appointments is recommended. Moreover, increasing awareness of the locations on and off campus that offer mental health services may assist students in finding a location better suited to their needs (Stebleton et al., 2014).

### 5.1 Limitations

As discussed earlier, I acknowledge that my own experiences with mental health and mental health service access lead me to conduct this research to explore ways to better support service access for emerging adults. Due to my own experiences, I found I was able to relate to some mental health and service use experiences that participants shared which contributed to my coding of the data. However, engaging in the process of reflexivity and taking a collaborative approach to this research helped to produce and develop themes that stayed true to the participant responses, rather than using a predetermined approach to coding.

It is important to note that the participants in this study primarily identified as cis‐women and white. Future research is needed to explore experiences in more diverse participants and population subgroups that comprise the student body, including consideration of ethnicity, gender, socioeconomic status, and first generation and international students. Relatedly, the research team was relatively homogenous, primarily including white women over the age of 28, which may contribute to bias within the analysis. However, the use of critical friends, that were in the final year of their undergraduate programs or had recently graduated, throughout the development and analysis process to challenge our assumptions is a strength of this study. Further, reflecting on prior experiences may be subject to prompt wording, memory, and social desirability biases (Bickart et al., 2006; Garry et al., 2002). To aid in recall, context was added before questions were asked (McKenna et al., 2004). Participants may have agreed to participate because they felt strongly about sharing a mental health service use experience they had, which may not reflect the experiences of the general student population but may highlight particularly relevant challenges and facilitators. Moreover, this study took place within the context of the COVID‐19 pandemic and some experiences may not be applicable to students graduating outside of this context, particularly the impact of the COVID‐19 pandemic theme. However, research during this period helps to underscore the importance of elements of the typical learning and graduation experience that were interrupted, such as convocation and connections with peers. During the pandemic, key developmental tasks for emerging adults were interrupted for students transitioning out of postsecondary education. For example, feeling in‐between was extended as graduating students faced a delay in moving towards an adult life, identity explorations were paused as social connections were interrupted and some students had to move back home with parents due to closures and/or job loss (Ruderman Family Foundation & Arnett, 2024). Future research should explore similarities and differences in graduating student experiences post‐pandemic. Finally, the study sample was limited to one postsecondary institution, and student experiences may differ across varying institutions.

## 6 CONCLUSION

Participants discussed feeling pressured to succeed, uncertainty, and unpreparedness for next steps, the importance of connections to peers and professors, a lack of motivation and feeling unfinished due to the COVID‐19 pandemic response, and the need for flexible and accessible mental health services to address immediate and longer‐term needs. Results align with key features in theories of emerging adulthood and have implications for how we can better support students as they prepare for graduation. Further research expanding to experiences postgraduation is warranted to best support positive transitions.

## CONFLICT OF INTEREST STATEMENT

The authors declare no conflicts of interest.

## ETHICS STATEMENT

This research was approved by the University Research Ethics Committee, and all procedures were conducted following ethical standards.

## Data Availability

The data set used during the current study are available from the corresponding author [MM], upon reasonable request. The data that support the findings of this study are available on request from the corresponding author. The data are not publicly available due to privacy or ethical restrictions.

## APPENDIX 1 INTERVIEW TRANSCRIPT

### Expectations & Challenges/University Support (during final year)

1. Context (to help participant recall/memory): For the next few questions, I want you to think about your final year of your undergraduate program at Brock, from starting your final year of your degree in September, all the way to your final exam or assignment in April.
2. What was that final year like for you in terms of preparing to graduate?
  a. Probe: How did your experiences align or differ from your expectations?
  b. Probe: What stands out to you as significant about that year, as opposed to the other years of your degree?
3. What were key challenges in your final year as you prepared to graduate?
  a. Probe: You don't have to share details if you're not comfortable, but it would be helpful to know if any external or personal challenges influenced your ability to plan/work towards your next steps after graduating.
4. What was helpful in the final year as you prepared to graduate?
  a. Probe: Looking back, would you have done anything differently as you approached graduation?
5. Is there anything that you wish had been available to support you in your final year?
  i. Probe: What services or support do you wish had been available?
6. The next few questions are specific to your mental health experiences and accessing mental health support.
  a. How did your experiences in preparing to graduate relate to your mental health?
7. Did you ever feel that you needed support for your mental health during your final year?
  a. If yes, but didn't seek support → What prevented you from reaching out for support?
  b. If yes and sought support → What was your experience like accessing mental health support during your final year?
  c. Probe: What types of support did you try to access?
  d. Probe: Did you try to access any campus mental health supports in your final year?
  e. Probe: Did you try to access mental health support off campus?
  f. Probe: Did you reach out to friends, family, roommates, or others?
  g. Probe: Was there anywhere or anyone that else that you reached out to for mental health support?
  h. Probe: Where did you find the most success in seeking support?
8. How helpful were the supports that you accessed?
  a. Probe: What made the support from [*ask for each type of support that participant indicated they received*] helpful or not helpful?
  b. Probe: What would make it more helpful?
9. Did you experience any barriers in accessing support? Can you tell me about them?
  a. Probe: What do you think would have supported you in accessing services?
  b. If no → Would you have known where to access support if needed, or to direct a friend to, if they had asked you where to get support?
10. Was there anything that you wish had been available in your final year to support your mental health?

### Expectations & Challenges/University Support (after final year)

1. Context: Now, for the next few questions, I want you to think about your time since graduating from your undergraduate program at Brock University, from that spring after you completed your final courses, and up until now.
2. How has the transition after graduating been for you?
  a. Probe: Thinking back to when you graduated, and your expectations of what the transition after graduating would be like, how have your experiences aligned or differed from what you expected?
3. How have your experiences (e.g., related to school, careers, finances, relationships, or other aspects of personal life) since graduating related to your mental health?
  a. Probe: Has graduating impacted your mental health in a positive or negative way? Tell me what you mean by negative and/or positive?
4. What are some key challenges that you've experienced in the transition from your undergraduate degree?
  a. Probe: Were there any *unexpected* challenges that you did not anticipate?
  b. Probe: You don't need to explain anything too sensitive, but it would be helpful to know if this was due to a particular unpredictable life event (like a family emergency, accident, or relationship change)? Or was it just something you didn't foresee about life after your undergraduate degree?
5. Have you ever felt that you needed support for your mental health since graduating? Have you tried to access support?
  a. If yes, but didn't seek support → What prevented you from reaching out to support?
  b. If yes and sought support → What was your experience like accessing mental health support since graduating?
  c. Probe: What types of support have you tried to access?
  d. Probe: Did you try to access any community supports?
  e. Probe: Where did you find the most success in seeking support?
  f. Did you experience anything that you found helpful when accessing support?
6. How helpful were the supports that you accessed?
  a. Probe: What made the support from [*ask for each type of support that participant indicated they received*] helpful or not helpful?
  b. Probe: What would make it more helpful?
7. Did you experience any barriers in accessing support? Can you tell me about them?
  a. Probe: Did you have trouble finding access to mental health support?
  b. Probe: What do you think would have supported you in accessing support?
  c. If no → Would you know where to seek support if needed, or to direct a friend to, if they had asked you where to get support?
8. Was there anything that you wish had been available to support you in the transition since graduating?

### Closing

Is there anything that we have missed that you would like to add about the transition out of university?

- Probe: Anything to add about how students could be supported for the transition?

Thank you for sharing you experiences. I am going to stop the recording now. We hope this research will help us prepare and support students in the transition out of university and wish you luck in your future endeavors!

## References

[jad12431-bib-0057] Akkermans, J. , Blokker, R. , Buers, C. , Heijden, B. V. , & Vos, A. D. (2021). Ready, set, go! School‐to‐work transition in the new career | Young adult development at the school‐to‐work transition international pathways and processes | Oxford Academic [Internet]. [cited 2023 Nov 20]. Available from: https://academic.oup.com/book/40024/chapter/340387296

[jad12431-bib-0001] Allan, G. (2008). Flexibility, friendship, and family. Personal Relationships, 15(1), 1–16.

[jad12431-bib-0070] Alonso, J. , Angermeyer, M. C. , Bernert, S. , Bruffaerts, R. , Brugha, T. S. , Bryson, H. , de Girolamo, G. , Graaf, R. , Demyttenaere, K. , Gasquet, I. , Haro, J. M. , Katz, S. J. , Kessler, R. C. , Kovess, V. , Lépine, J. P. , Ormel, J. , Polidori, G. , Russo, L. J. , Vilagut, G. , … Vollebergh, W. A. M. (2004). Use of Mental Health Services in Europe: Results from the European Study of the Epidemiology of Mental Disorders (ESEMeD) project—2004—Acta Psychiatrica Scandinavica ‐ Wiley Online Library [Internet]. [cited 2023 Oct 18]. Available from: https://onlinelibrary-wiley-com.proxy.library.brocku.ca/doi/full/10.1111/j.1600-0047.2004.00330.x 10.1111/j.1600-0047.2004.00330.x 15128387

[jad12431-bib-0002] Arnett, J. J. (2011). Emerging adulthood(s): The cultural psychology of a new life stage, In: Bridging cultural and developmental approaches to psychology: New syntheses in theory, research, and policy. New York, NY, US (pp. 255–275). Oxford University Press.

[jad12431-bib-0003] Arnett, J. J. (2014). Emerging adulthood: The winding road from the late teens through the twenties [Internet]. Oxford University Press. [cited 2023 Nov 15]. Available from: https://academic.oup.com/book/26128

[jad12431-bib-0004] Arnett, J. J. (2015). The Oxford handbook of emerging adulthood (p. 657). Oxford University Press.

[jad12431-bib-0005] Arnett, J. J. , Žukauskienė, R. , & Sugimura, K. (2014). The new life stage of emerging adulthood at ages 18–29 years: Implications for mental health. The Lancet Psychiatry, 1(7), 569–576. 26361316 10.1016/S2215-0366(14)00080-7

[jad12431-bib-0006] Bengtsson, M. (2016). How to plan and perform a qualitative study using content analysis. NursingPlus Open, 2, 8–14.

[jad12431-bib-0007] Berger, R. (2015). Now I see it, now I don't: Researcher's position and reflexivity in qualitative research. Qualitative Research, 15(2), 219–234.

[jad12431-bib-0008] Bickart, B. A. , Phillips, J. M. , & Blair, J. (2006). The effects of discussion and question wording on self and proxy reports of behavioral frequencies. Marketing Letters, 17(3), 167–180.

[jad12431-bib-0009] Braun, V. , & Clarke, V. (2013). Successful qualitative research: A practical guide for beginners (p. 402). SAGE.

[jad12431-bib-0010] Brown, S. , Parker, J. , & Godding, P. , Administrative, clinical, and ethical issues surrounding the use of waiting lists in the delivery of mental health services | SpringerLink [Internet]. [cited 2023 Oct 25]. Available from: https://link.springer.com/article/10.1007/BF02287708 10.1007/BF02287708 12032979

[jad12431-bib-0011] Brzinsky‐Fay, C. , & Solga, H. (2016). Compressed, postponed, or disadvantaged? School‐to‐work‐transition patterns and early occupational attainment in West Germany. Research in Social Stratification and Mobility, 46, 21–36.

[jad12431-bib-0012] Carver, J. , Cappelli, M. , & Davidson, S. (2015). Taking the next step forward [Internet]. Mental Health Commission of Canada. [cited 2022 May 3]. Available from: http://public.ebookcentral.proquest.com/choice/publicfullrecord.aspx?p=4383709

[jad12431-bib-0013] Cebulla, A. , & Whetton, S. (2018). All roads leading to Rome? The medium term outcomes of Australian youth's transition pathways from education. Journal of Youth Studies, 21(3), 304–323.

[jad12431-bib-0067] Centre for Innovation in Campus Mental Health (CICMH). (2019). Stepped Care Guide V13 [Internet]. [cited Oct 23]. Available from: https://campusmentalhealth.ca/wp-content/uploads/2019/09/Stepped-Care-Guide-V13.pdf

[jad12431-bib-0014] Chrikov, I. , Soria, K. M. , Horgos, B. , & Jones‐White, D. (2020). Undergraduate and graduate students' mental health during the COVID‐19 pandemic [Internet]. SERU Consortium Reports, University of California ‐ Berkeley and University of Minnesota, 1, 1–10. http://conservancy.umn.edu/handle/11299/215271

[jad12431-bib-0015] Collins, S. R. , Schoen, C. , Kriss, J. L. , Doty, M. M. , & Mahato, B. (2005). Rite of passage? Why young adults become uninsured and how new policies can help. Commonw Fund Issue Briefs [Internet], 1(20), 1–4. May 1 [cited 2023 Oct 18]. Available from: http://www.cmwf.org/publications/publications_show.htm?doc_id=374136 16830442

[jad12431-bib-0062] Creswell, J. W. , & Poth, C. N. (2023). Qualitative inquiry and research design. SAGE Publications Inc . [Internet]. [cited 2023 Sep 5]. Available from: https://us.sagepub.com/en-us/nam/qualitative-inquiry-and-research-design/book246896

[jad12431-bib-0016] Deci, E. L. , La Guardia, J. G. , Moller, A. C. , Scheiner, M. J. , & Ryan, R. M. (2006). On the benefits of giving as well as receiving autonomy support: Mutuality in close friendships. Personality and Social Psychology Bulletin, 32(3), 313–327. 16455859 10.1177/0146167205282148

[jad12431-bib-0017] Dotson, M. P. , Castro, E. M. , Magid, N. T. , Hoyt, L. T. , Suleiman, A. B. , & Cohen, A. K. (2022). “Emotional distancing”: Change and strain in U.S. young adult college students' relationships during COVID‐19. Emerging Adulthood, 10(2), 546–557. 35382514 10.1177/21676968211065531 PMC8919111

[jad12431-bib-0018] Downe‐Wamboldt, B. (1992). Content analysis: Method, applications, and issues. Health Care for Women International, 13(3), 313–321. 1399871 10.1080/07399339209516006

[jad12431-bib-0019] Eimers, M. T. (2001). The impact of student experiences on progress in college: An examination of minority and nonminority differences. NASPA Journal, 38(3), 386–409.

[jad12431-bib-0020] Eisenberg, D. , Gollust, S. E. , Golberstein, E. , & Hefner, J. L. (2007). Prevalence and correlates of depression, anxiety, and suicidality among university students. American Journal of Orthopsychiatry, 77(4), 534–542. 18194033 10.1037/0002-9432.77.4.534

[jad12431-bib-0021] Elo, S. , Kääriäinen, M. , Kanste, O. , Pölkki, T. , Utriainen, K. , & Kyngäs, H. (2014). Qualitative content analysis: a focus on trustworthiness. Sage Open, 4(1), 2158244014522633.

[jad12431-bib-0022] Fusch, P. , & Ness, L. (2015). Are we there yet? Data saturation in qualitative research. The Qualitative Report, 20(9), 1408–1416.

[jad12431-bib-0023] Garry, M. , Sharman, S. J. , Feldman, J. , Marlatt, G. A. , & Loftus, E. F. (2002). Examining memory for heterosexual college students' sexual experiences using an electronic mail diary. Health Psychology, 21(6), 629–634. 12433018 10.1037//0278-6133.21.6.629

[jad12431-bib-0024] Goodell, L. S. , Stage, V. C. , & Cooke, N. K. (2016). Practical qualitative research strategies: Training interviewers and coders. Journal of Nutrition Education and Behavior, 48(8), 578–585.e1. 27395426 10.1016/j.jneb.2016.06.001

[jad12431-bib-0025] Grabau, L. “Academic Motivation and Student Development During the Transition to C” [Internet]. [cited 2024 Aug 1]. Available from: https://encompass.eku.edu/kjectl/vol7/iss1/1/

[jad12431-bib-0026] Grosemans, I. , Hannes, K. , Neyens, J. , & Kyndt, E. (2020). Emerging adults embarking on their careers: Job and identity explorations in the transition to work. Youth & Society, 52(5), 795–819.

[jad12431-bib-0027] Halfon, N. , Forrest, C. B. , Lerner, R. M. , & Faustman, E. M. editors. (2018). Handbook of life course health development [Internet]. Springer International Publishing. [cited 2022 May 3]. Available from: http://link.springer.com/10.1007/978-3-319-47143-3

[jad12431-bib-0028] Hallett, R. E. , Kezar, A. , Perez, R. J. , & Kitchen, J. A. (2020). A typology of college transition and support programs: Situating a 2‐year comprehensive college transition program within college access. American Behavioral Scientist, 64(3), 230–252.

[jad12431-bib-0029] Halstead, T. J. (2014). An exploration of career transition self‐efficacy in emerging adult college graduates [Internet] [D.Ed.]. [United States—Pennsylvania]. Indiana University of Pennsylvania. [cited 2023 Nov 15]. Available from: https://www.proquest.com/docview/1640768834/abstract/A559792C66BA4286PQ/1

[jad12431-bib-0030] Halstead, T. J. (2021). Requisite metamorphoses: college graduates transitioning from school to work. In E. A. Marshall , & J. E. Symonds editors, Young adult development at the school‐to‐work transition: International pathways and processes. Oxford University Press. [cited 2023 Nov 20]. p. 0. Available from: 10.1093/oso/9780190941512.003.0020

[jad12431-bib-0031] Huegaerts, K. , Wagener, M. , & Vanroelen, C. (2020). Is mental health a predictor for a smooth school‐to‐work‐transition? A 20‐month follow‐up study of Brussels youth. Applied Research in Quality of Life, 15(5), 1549–1567.

[jad12431-bib-0032] Hutchison, E. D. (2011). Life course theory. In: R. J. R. Levesque editor, Encyclopedia of adolescence [Internet] (pp. [cited 2022 Aug 22]. 1586–1594). Springer. Available from: 10.1007/978-1-4419-1695-2_13

[jad12431-bib-0033] Johnson, M. , Berg, J. , & Sirotzki, T. , Differentiation in self‐perceived adulthood: Extending the confluence model of subjective age identity, 2007 [Internet]. [cited 2023 Oct 27]. Available from: https://journals.sagepub.com/doi/abs/10.1177/019027250707000304?casa_token=bSI8ECyarz0AAAAA:df6TwquNAZoDVTTiTf9qM0R_3Pa24Pd-DdSRSFslpJ_pFuDxre0fotWhpAP97C3K9WA3w1-CzShKTg

[jad12431-bib-0034] Kindelberger, C. , Safont‐Mottay, C. , Lannegrand‐Willems, L. , & Galharret, J. Searching for autonomy before the transition to higher education: How do identity and self‐determined academic motivation co‐evolve? Journal of Youth and Adolescence [Internet]. [cited 2024 Aug 1]. Available from: https://link.springer.com/article/10.1007/s10964-019-01137-5 10.1007/s10964-019-01137-5 31598810

[jad12431-bib-0035] Koivisto, P. , Vuori, J. , & Vinokur, A. D. (2010). Transition to work: Effects of preparedness and goal construction on employment and depressive symptoms. Journal of Research on Adolescence, 20(4), 869–892.

[jad12431-bib-0036] Komarraju, M. , Musulkin, S. , & Bhattacharya, G. (2010). Role of student–faculty interactions in developing college students' academic self‐concept, motivation, and achievement. Journal of College Student Development, 51(3), 332–342.

[jad12431-bib-0037] Koo, K. , Kim, Y. W. , Lee, J. , & Nyunt, G. (2021). “It's my fault”: Exploring experiences and mental wellness among Korean international graduate students. Journal of International Students [Internet], 11(4), 790–811. Available from: https://ojed.org/index.php/jis/article/view/2801

[jad12431-bib-0038] Kuh, G. D. , & Hu, S. (2001). The effects of student‐faculty interaction in the 1990s. The Review of Higher Education, 24(3), 309–332.

[jad12431-bib-0039] Lambert, N. M. , Stillman, T. F. , Hicks, J. A. , Kamble, S. , Baumeister, R. F. , & Fincham, F. D. , To belong is to matter: Sense of belonging enhances meaning in life. 2013 [Internet]. [cited 2023 Oct 31]. Available from: https://journals.sagepub.com/doi/abs/10.1177/0146167213499186 10.1177/0146167213499186 23950557

[jad12431-bib-0040] Larcombe, W. , Finch, S. , Sore, R. , Murray, C. M. , Kentish, S. , Mulder, R. A. , Lee‐Stecum, P. , Baik, C. , Tokatlidis, O. , & Williams, D. A. (2016). Prevalence and socio‐demographic correlates of psychological distress among students at an Australian university. Studies in Higher Education, 41(6), 1074–1091.

[jad12431-bib-0041] MacDonald, K. , Fainman‐Adelman, N. , Anderson, K. K. , & Iyer, S. N. (2018). Pathways to mental health services for young people: A systematic review. Social Psychiatry and Psychiatric Epidemiology, 53(10), 1005–1038. 30136192 10.1007/s00127-018-1578-y PMC6182505

[jad12431-bib-0042] MacLeod, K. B. , & Brownlie, E. B. (2014). Mental health and transitions from adolescence to emerging adulthood: Developmental and diversity considerations. Canadian Journal of Community Mental Health, 33(1), 77–86.

[jad12431-bib-0043] Magier, M. J. , Law, M. , Pennisi, S. , Martini, T. , Duncan, M. J. , Chattha, H. , & Patte, K. A. (2023). Final‐year university students' mental health and access to support as they prepared to graduate. Cogent Mental Health, 2(1), 2252918. 10.1080/28324765.2023.2252918 PMC12442996 41262368

[jad12431-bib-0044] Marler, E. K. , Bruce, M. J. , Abaoud, A. , Henrichsen, C. , Suksatan, W. , Homvisetvongsa, S. , & Matsuo, H. (2021). The impact of COVID‐19 on university students' academic motivation, social connection, and psychological well‐being. Scholarship of Teaching and Learning in Psychology [Internet].[cited 2023 Oct 31]; Available from: 10, 320–330. 10.1037/stl0000294

[jad12431-bib-0045] Marshall, E. A. , & Symonds, J. E. (2021). Young adult development at the school‐to‐work transition: International pathways and processes (p. 529). Oxford University Press.

[jad12431-bib-0064] Marshall, E. A. , & Butler, K. (2014). School‐to‐work transitions in emerging adulthood | The Oxford handbook of emerging adulthood. Oxford Academic. [Internet]. [cited 2023 Nov 20]. Available from: https://academic.oup.com/edited-volume/28014/chapter/211806637?searchresult=1

[jad12431-bib-0072] Mental Health Commission of Canada (MHCC). (2021). What is Stepped Care 2.0 [Internet]. [cited 2023 Feb 23]. Available from: https://mentalhealthcommission.ca/wp-content/uploads/2021/11/What-is-Stepped-Care-2.0-4-pager.pdf

[jad12431-bib-0046] McGorry, P. D. , & Mei, C. (2018). Early intervention in youth mental health: Progress and future directions. Evidence‐Based Mental Health, 21(4), 182–184. 30352884 10.1136/ebmental-2018-300060 PMC10270418

[jad12431-bib-0047] McKenna, J. , Foster, L. J. , & Page, A. (2004). Exploring recall of physical activity in young people using qualitative interviewing. Pediatric Exercise Science, 16(1), 5–14.

[jad12431-bib-0048] McNamara, B. C. , Madsen, S. D. , & DeGrace, A. (2015). Growing Up with a Little Help from their friends in emerging adulthood. In J. J. Arnett editor, The Oxford handbook of emerging adulthood [Internet]. Oxford University Press. [cited 2023 Oct 25]. p. 0. Available from: 10.1093/oxfordhb/9780199795574.013.008

[jad12431-bib-0049] Micari, M. , & Pazoz, P. (2012). Connecting to the professor: impact of the student–faculty relationship in a highly challenging course [Internet]. [cited 2023 Nov 16]. Available from https://www.tandfonline.com/doi/full/10.1080/87567555.2011.627576

[jad12431-bib-0050] Molgat, M. (2007). Do transitions and social structures matter? How “emerging adults” define themselves as adults. Journal of Youth Studies, 10(5), 495–516.

[jad12431-bib-0051] Murphy, K. A. , Blustein, D. L. , Bohlig, A. J. , & Platt, M. G. (2010). The college‐to‐career transition: An exploration of emerging adulthood. Journal of Counseling & Development, 88(2), 174–181.

[jad12431-bib-0052] Osuna, E. E. (1985). The psychological cost of waiting. Journal of Mathematical Psychology, 29(1), 82–105.

[jad12431-bib-0053] Pascarella, E. T. , & Terenzini, P. T. (1979). Student‐faculty informal contact and college persistence: A further investigation. The Journal of Educational Research, 72(4), 214–218.

[jad12431-bib-0054] Perry, A. L. Treading through swampy water: Graduates' experiences of the post‐university transition. (2012). [cited 2023 May 24]; Available from: https://ir.canterbury.ac.nz/handle/10092/10343

[jad12431-bib-0055] Pfeiffer, D. Academic and environmental stress among undergraduate and graduate college students: a literature review.

[jad12431-bib-0056] Philpot, L. M. , Ramar, P. , Roellinger, D. L. , Barry, B. A. , Sharma, P. , & Ebbert, J. O. (2021). Changes in social relationships during an initial “stay‐at‐home” phase of the COVID‐19 pandemic: A longitudinal survey study in the U.S. Social Science & Medicine, 274, 113779. 33639395 10.1016/j.socscimed.2021.113779 PMC7895700

[jad12431-bib-0061] Ravitch, S. M. , & Carl, N. M. (2022). Qualitative research. SAGE Publications Inc . [Internet]. [cited 2023 Jun 29]. Available from: https://us.sagepub.com/en-us/nam/node/60839/print

[jad12431-bib-0058] Rogers, A. A. , Ha, T. , & Ockey, S. (2021). Adolescents' perceived socio‐emotional impact of COVID‐19 and implications for mental health: Results from a U.S.‐based mixed‐methods study. Journal of Adolescent Health, 68(1), 43–52. 10.1016/j.jadohealth.2020.09.039 PMC7605752 33143986

[jad12431-bib-0059] Rudenstine, S. , McNeal, K. , Schulder, T. , Ettman, C. K. , Hernandez, M. , Gvozdieva, K. , & Galea, S. (2021). Depression and anxiety during the COVID‐19 pandemic in an urban, low‐income public university sample. Journal of Traumatic Stress, 34(1), 12–22. 33045107 10.1002/jts.22600 PMC7675401

[jad12431-bib-0060] Ruderman Family Foundation . Arnett, J. The mental health effects of COVID‐19‐ a continuing crisis, especially for emerging adults ages 18–29 [Internet]. [cited 2024: Aug 21]. Available from https://rudermanfoundation.org/white_papers/the-mental-health-effects-of-covid-19-a-continuing-crisis-especially-for-emerging-adults-ages-18-29/

[jad12431-bib-0063] Schaefer, S. M. , Morozink Boylan, J. , van Reekum, C. M. , Lapate, R. C. , Norris, C. J. , Ryff, C. D. , & Davidson, R. J. (2013). Purpose in life predicts better emotional recovery from negative stimuli. PLoS One, 8(11), e80329. 24236176 10.1371/journal.pone.0080329 PMC3827458

[jad12431-bib-0065] Smith, B. , & McGannon, K. R. (2018). Developing rigor in qualitative research: Problems and opportunities within sport and exercise psychology. International Review of Sport and Exercise Psychology, 11(1), 101–121.

[jad12431-bib-0066] Stebleton, M. J. , Soria, K. M. , & Huesman, Jr. R. L. (2014). First‐generation students' sense of belonging, mental health, and use of counseling services at public research universities. Journal of College Counseling, 17(1), 6–20.

[jad12431-bib-0068] Tamminen, K. A. , & Poucher, Z. A. (2020). Research philosophies. In: D. Hackfort , & R. Schinke editors, The Routledge international encyclopedia of sport and exercise psychology [Internet] (1st ed, pp. [cited 2022 Aug 22]. 535–549). Routledge. Available from: https://www.taylorfrancis.com/books/9781351739559/chapters/10.4324/9781315187259-39.

[jad12431-bib-0069] Umbach, P. D. , & Wawrzynski, M. R. (2005). Faculty do matter: the role of college faculty in student learning and engagement. Research in Higher Education, 46(2), 153–184.

[jad12431-bib-0071] Weber, R. (1990). Basic content analysis [Internet]. SAGE Publications, Inc. [cited 2023 Oct 16]. Available from: https://methods.sagepub.com/book/basic-content-analysis

[jad12431-bib-0073] Wood, D. , Crapnell, T. , Lau, L. , Bennett, A. , Lotstein, D. , Ferris, M. , & Kuo, A. (2017). Emerging adulthood as a critical stage in the life course. In: Handbook of life course health development, 1, 123–143. 31314293

[jad12431-bib-0074] Yang, N. , & Yang, X. (2022). Anxiety and depression in graduating university students during the COVID‐19 pandemic: A longitudinal study. American Journal of Translational Research, 14(4), 2668–2676. 35559421 PMC9091131

[jad12431-bib-0075] Yeager, D. , Walton, G. , & Cohen, G. L. (2013). Addressing achievement gaps with psychological interventions. Phi Delta Kappan, 94(5), 62–65.

